# Hip Abductor Strengthening Improves Physical Function Following Total Knee Replacement: One-Year Follow-Up of a Randomized Pilot Study

**DOI:** 10.2174/1874312901711010030

**Published:** 2017-03-31

**Authors:** Karvannan Harikesavan, Raj D. Chakravarty, Arun G Maiya, Sanjay P. Hegde, Shivakumar Y. Shivanna

**Affiliations:** 1 Department of Physiotherapy, School of Allied Health sciences, Manipal University, Bangalore. India.; 2 Orthopaedic joint replacement surgeon. Manipal Hospital, Bangalore. India.; 3 Department of Physiotherapy, School of Allied Health Sciences, Manipal University, Manipal, India.

**Keywords:** Total knee replacement, Hip abductor strengthening, Knee exercises, Single leg stance, Osteoarthritis

## Abstract

**Background:**

Total knee replacement (TKR) is the commonest surgical procedure for patients with severe pain and impaired physical function following end stage knee osteoarthritis. The hip abductors are well renowned in stabilization of the trunk and hip during walking, maintaining the lower limb position, and transferring the forces from the lower limbs to the pelvis.

**Objective:**

To assess the efficacy of hip abductor strengthening exercise on functional outcome using performance based outcome measures following total knee replacement.

**Methods:**

An observer blinded randomized pilot trial design was conducted at Manipal hospital, Bangalore, India. Participants designated for elective TKR were randomized to experimental group hip abductor strengthening along with standard rehabilitation (n=10) or control group standard rehabilitation alone (n=10). Participants followed for one year to assess physical function using performance based outcomes, such as timed up and go test, single leg stance test, six minute walk test, knee extensor strength and hip abductor strength.

**Result:**

Eighteen participants with a mean age of 63.1 ± 5.5 years (8 Males and 10 Females) completed the study. Improvement in hip abduction strength, single leg stand test was superior in hip abductor strengthening group at 3 months and 1 year when compared to standard rehabilitation alone.

**Conclusion:**

Hip abductor strengthening showed superior improvements in single leg stance test and six minute walk test. Hip abductor strengthening exercises has the potential to improve physical function following total knee replacement.

## INTRODUCTION

Total knee replacement (TKR) is the most renowned surgical procedure for patients with severe knee pain and impaired function following end stage knee osteoarthritis [1, 2]. Significant improvements in pain reduction, improved function and patient satisfaction were the main expectations following TKR [3]. Studies reported that participants showed substantial improvements in arthritis pain, but a varied physical function following TKR [4, 5].

Despite more advanced and excellent surgical procedure, declined functional tasks were reported following TKR when compared to the healthy age matched controls with 15% reduced walking speed, 50% more time taken to complete stair climbing tasks and 20% less distance covered during the six minute walk test [6, 7]. Franklin *et al*. [3], suggested that demographic and clinical variables would predict the functional improvement especially the quadriceps strength. The strengthening of the quadriceps muscles was established by previous studies and proved its short-term improvements in physical function after TKR [8-10].

A systematic review of Minne lowis *et al*. [11], and Artz *et al*. [12], recommended that future trials should tailor the post-operative interventions for enhanced functional performance measures and long-term benefits. Arnold *et al*. in their recent systematic review found the long-term changes in physical activity following TKR, but with limited evidence and recommended to reiterating the approaches to improve the physical function and patients’ expectations following TKR [13].

Reconnoitering the modifiable factors contributing to the functional performances following TKR might help to improve performance based outcomes, one such modifiable factor is the lower extremity muscle weakness following knee osteoarthritis (OA). A recent systematic review and meta-analysis of symptomatic knee OA patients revealed the weakness of isometric and isokinetic hip abductor strength [14]. Recent studies on total knee arthroplasty revealed that hip abductor strength was positively correlated to quadriceps strength for enhanced performance-based function measures [15, 16]. The hip abductors are well renowned as a primary muscle group for the stabilization of trunk and hip during walking, maintaining the femoropelvic alignment, femoral head stability and transferring the forces from the lower limbs to the pelvis [17-19]. Moreover, weakness of the hip abductor resulting in poorer functional performance in older adults and associated with reduced physical performance in patients with knee OA [20]. Hence, we hypothesized that the hip abductor strengthening might augment physical function in participants post TKR.

## MATERIALS AND METHODOLOGY

### Study Design

We conducted an observer blinded, randomized pilot study with outcome measurement taken before surgery and at 1 month, 3 months and 12 months following TKR. The study was conducted at College of Physiotherapy, School of Allied Health Sciences, Manipal hospital, Bangalore, India. The study protocol was approved by the Institutional Research Committee of School of Allied Health Sciences, Manipal University, Manipal, India.

### Participants

Participants posted for the elective TKR screened for the inclusion and exclusion criteria. Participants were included if 50 years of age or older with osteoarthritis as diagnosis of the either knee or both knees was painful and they specified the most painful knee. All the study participants were screened by the orthopaedic surgeons for the diagnosis and staging of knee osteoarthritis based on the major elements of the diagnostic criteria like history, physical examination, imaging studies [21, 22]. The participants who were diagnosed with end stage knee osteoarthritis were posted for elective total knee replacement.

They were excluded, if diagnosed other than knee osteoarthritis, any neurological impairment that may alter the lower extremity performance, any other orthopedic surgery in either leg in the past year. All eligible participants signed an informed consent. Using block randomization, participants were randomly allocated to either experimental or control group. Prior to the enrollment of the participants, the Institutional research committee of Manipal University approved the study. The rights of the included participants have been reserved..

A total of 20 participants were included and randomly assigned to hip abductor strengthening group (n=10) or the control group (n=10) using block randomization prior to the surgery. A total of 10 males (6 in hip abductor strengthening group and 4 in the control group) and 10 females (4 in hip abductor strengthening group and 6 in the control group) were included in the study with a mean age of 63.3 (5.4) in hip abductor strengthening group and 62.8 (5.9) in the control group. The mean body mass index (BMI) of the hip abductor strengthening group was 26.5 (3.2) and the control group was 29.8 (3.2), respectively.

### Rehabilitation Protocol

The rehabilitation protocol was established by the authors, physiotherapy department, Manipal University, Bangalore, India. All the participants in the control group underwent a standard rehabilitation protocol from day of operation until discharge. The standard rehabilitation focused on early mobilization, such as reducing pain and swelling, improving knee flexion and extension range of motion, and progressive quadriceps strengthening exercises to maximize function (Appendix-1). Hip abductor strengthening exercise group underwent hip abductor strengthening exercises along with the standard rehabilitation (Appendix-2). Both the groups underwent 30- 40 minutes of supervised physical therapy per session, twice a day until discharge. Following discharge the participants underwent supervised rehabilitation for 4-5 sessions in a week over 4 weeks duration, and thereafter 2-3 sessions per week for 12 weeks with each session lasted for about 40- 45 minutes. They were asked to continue their home based exercise and the exercise adherence was ensured through logbook and periodic telephone calls.

### Variables

Baseline outcome measures of timed up and go test (TUG), six minute walk test (SMWT), single leg stance (SLS), numeric pain rating scale (NPRS), knee extensor strength and hip abductor strength were taken prior to the surgery. Post outcome measure of the same was recorded at 1 month, 3 months and 1 year following the surgery. An independent blinded observer with more than 5 years of experience in physical therapy collected the outcome measures.

### Physical Function

Physical function allied to the person's ability to move around for performing the activities of daily living [23]. It can be measured using the performance based tests or self- reported measures using the questionnaires, both measures quarantine different forms of physical functioning [24-26]. The performance based measures were often assessed through observing the number of repetitions, timing or the totaling distance covered. These measures ideally evaluate the performance of the participants rather than the perception of what they can do [23, 25]. Performance based tests might evaluate distinguishing pain and function other than familiar self-reported questionnaires [25, 26]. However, studies have revealed that both the measure is seen as amenable, rather than challenging when appraising physical function for patients with hip and knee OA also follows total knee replacement [26-28].

### Timed Up and Go Test

Timed up-and-go test (TUG) also termed as the ambulatory transitions test used for identifying problems in functional mobility [29-31]. The TUG comprises various activities, such as sit-to-stand from a standard chair with armrest (46 cm seat height), walking 3 m distance in their usual manner (customary walking aids if necessary), and turning at the end while walking and back to their chair to sit down again [32, 33]. With regular footwear, all the participants underwent one warm up trial prior to being timed with fastest of two trials [29]. TUG has an excellent inter-rater and intra-rater reliability in older adults and is responsive to changes after TKR and it has the ability to distinguish the physical functional performance of healthy subjects from patients with TKR [24, 32, 33].

### Six Minute Walk Test

The six minute walk test (SMWT) assesses the physical function by totaling the distance covered maximally by the participant walking at their free speed in patients with knee osteoarthritis and people who are indicated for surgery [24, 34-37]. Participants asked to walk as quickly as they felt safe on a measured 46 meter uncarpeted rectangular indoor circuit during the 6-minute period. The participants walked as much distance as possible with an assistive device if required and the distance covered was measured to the nearest meter [35, 38].

### Single Leg Stance

Single leg stance (SLS) used to assess the static postural control during standing on the operated leg. The participants were asked to balance on their stance leg with hands on the hips and the test duration was limited to 30 Sec, the longest duration of the three trials was recorded, followed by one warm up trial. The test was discontinued when the swing leg touches the stance limb or the floor for balance, the stance foot displaced from the ground, or when the participants moved his arm away from hips. This test has shown to be reliable, responsive to interventions and it is a common clinical assessment tool for determining the functional ability of the participants of various ages and functional levels [39-42].

### Muscle Strength Test

Participants underwent hip abductor and knee extensor maximal isometric strength assessment using hand held dynamometer (HHD) (Fabricatio enterprises incorporation, New York). They were positioned in supine lying with the tested limb in neutral position for hip abductor measurement. The HHD placed 5 cm above the knee joint line of the tested limb and the non-tested limb was positioned in neutral for stability. Participants asked to abduct their tested lower limb as hard as they can for 5 seconds with the therapist holding the force transducer of the HHD. The participants were encouraged verbally during the test for their maximal effort; and the maximum volitional force generated by the participant was quantified hip abductor muscle strength in pounds. Knee extensor maximal isometric strength (Quadriceps) of the participants was assessed in sitting position with hip in 90° and knee in 70° of flexion. They performed knee extension as hard as they can of the tested limb with pelvis and trunk stabilized [43-46].

Single blinded assessor conducted all the test and retest measurement with three trials performed per side, followed by one warm up trial. The maximal volitional force generated on hip abductors and knee extensors quantified muscle strength in pounds. This method provides the objective and quantitative measurements of the hip abductor and knee strength and showed to be a valid and reliable clinical tool to test muscle strength for athletes, patients with knee OA and those who underwent TKR [44-46].

### Numeric Pain Rating Scale (NPRS)

The participants are asked to rate their pain intensity on an 11-point numeric pain rating scale (NPRS) where '0' indicates no pain and '10' indicates the worst pain imaginable. An independent blinded observer recorded the knee pain of the participant at baseline, 1 month, 3 months and 1 year following the surgery.

### Statistical Analysis

Statistical analysis was conducted using SPSS software (version 16.0; SPSS. Chicago, IL, USA). Repeated measures ANOVA was used to determine the effects of hip abductor strengthening and standard rehabilitation post total knee replacement at baseline, 1 month, 3 months and 1 year, respectively. Post-hoc analysis using Bonferroni's test was used to determine the pairwise comparisons at different measurement levels amongst experimental and control groups. Equality of variance for the continuous variables was tested using Leven’s test. The statistical significance was set at p value < 0.05 with 95% confidence intervals, and all the tests were 2-tailed.

## RESULTS

Eighteen participants (8 males and 10 females) with a mean age of 63.1 ± 5.5 years completed the study. Both groups were comparable on clinical and demographic characteristics at baseline, and also for functional measures of TUG, SMWT, SLS, NPRS, and hip and knee strength measurements. The baseline characteristics of the included participants are mentioned in (Table **1**). Two participants in the control group were not able to follow-up due to immediate post-operative complications. The pre-operative baseline score and changes within group at 1 month, 3 months, 1 year of knee extension strength, hip abductor strength, TUG, SMWT, SLS and NPRS are mentioned in (Table **2**). The comparison between the groups of all the outcome variables is mentioned in (Table **3**).

The Hip abductor strengthening group (HAS) has shown statistically significant changes in knee strength at 3 months when compared to baseline and the improvements were sustained during 1 year follow-up. The HAS group showed a significant change from 36.1 ± 8.4 to 40.9 ± 10.1 at 3 months and preserved till 1 year 41.7 ± 7.5. Knee strengthening group or the control group (KS) showed statistical significant changes from 34.8 ± 4.9 to 42.3 ± 4.3 at 3 months and 42.8 ± 3.1 at 1 year respectively. Both the groups did not show statistical significant changes at 1 month following TKR. Between group comparison using post hoc analysis did not show a statistical significant at all the post outcome measures.

Hip abductor strength test at baseline in the HAS group was 36.1 ± 6.0 and control group was 36 ± 7. The strength in HAS showed significant improvement at 1 month, 3 months and year with a value of 40.3 ± 7.2, 42.6 ± 6.9 and 45.7 ± 6.7, respectively. The control group showed a significant difference from baseline 36 ± 7 to 39.8 ± 6.2 at 1 year. Between groups comparison showed a statistically significant strength difference in the HAS group at 3 months and 1 year following TKR.

From the baseline to 1 month, both the groups had spent more duration while performing TUG. Both the groups showed the statistically significant improvements in one year following TKR. Between groups comparison on TUG, the functional task at all the levels did not show a superior change. The time taken to complete the TUG was reduced from 16.3 ± 4.2 from baseline to 11.3 ± 2.2 at 1 year follow-up for HAS group and control group has improved from 18 ± 4.3 to 13.8 ± 2.6.

The HAS group walked faster than the control group at 1 year duration during SMWT. The HAS group showed a significant change from a baseline value of 255.1 ± 79.2 meters to 387.6 ± 119.5 and to 474.1 ± 160.5 meters at 3 months and 1 year, respectively. The control group showed the improvement from a baseline value of 201.1 ± 64.4 to 380.6 ± 124.7 at 1-year duration. Between groups analysis showed a significant mean difference in SMWT at one year in HAS group with a p value of <0.002.

The test for static postural control was assessed using SLS and it showed improvement in HAS group at 3 months and 1 year duration. However, it was not statistically significant at 1 month duration. The HAS showed changes in SLS from a baseline value of 8.3 ± 4.5 to 13.7 ± 5.4 at 3 months and 15.2 ± 5.9 Sec at 1 year duration. Between group analysis revealed a superior improvements in the HAS group at 3 months and 1 year as compared to the control group.

The NPRS showed a statistically significant difference in both the groups at all the periods. The HAS group showed a significant difference form a baseline value of 7.1 ±.56 to 4.2 ± 1.1, 1.7 ± 0.6, 1.4 ± 0.9 at 1 month, 3 months and 1 year, respectively. The control group showed a significant improvement with 3.6 ± 1.5, 2.1 ±.8, 1.1 ± .8 at 1 month, 3 months and 1 year when compared to a baseline value of 6.5 ± 1.5. Between groups analysis did not find a significant difference in NPRS during all the period.

## DISCUSSION

The primary aim of this study is to determine whether hip abductor strengthening would improve physical function following total knee replacement when compared to standard rehabilitation. Hip abductor strengthening group showed a statistically significant improvement in physical functional measure, such as single leg stance at 3 months and 1 year and six-minute walk test at 1 year following TKR.

At one month and 3 months, the time taken to complete the TUG was not statistically significant and the participants in both groups showed an increased time to complete the task. Greater deficits were shown in the performance at 1 month and recovered to the preoperative levels at 3 months and it further showed significant gains at 1 year following surgery. Similarly, previous studies showed poor performances following TKR [7, 10] and this could possibly be due to the decreased quadriceps and hip abductor strength at one month following surgery.

The participants in the HAS group walked faster and longer than the KS participants at 1 year. The HAS group walked additional 132 meters at 3 months, 219 meters at 1 year and the KS group walked 118 meters more at 3 months and 179 meters at 1 year. The group's analysis has shown a significant difference at 1 year with a mean difference of 88.3 meters. A study targeting functional rehabilitation by Moffet *et al*. [10], found that their subjects walked 145 meters lesser at 12 months duration in SMWT following TKR. Petterson *et al*. [9], in their study found that the study patients walked 150 meters further 12 months post-operatively, our study participants performed additional 219 meters during SMWT, and these improvements might be attributed to the increased strength of hip abductors so as to provide the lateral stability of the pelvis during walking.

Single leg stance on the surgical side had decreased at one month in both the groups and the HAS group has shown a statistically and clinically significant differences at 3 months and 1 year duration. Piva *et al*. [15], observed a 25% difference in SLS between groups at 6 months following TKR on the surgical side in comparison our study between the group analysis has shown a mean difference of 3.9 Sec at 1 year. This could possibly due to the strength increments of the hip abductor muscle, augmentations of strength may enhance the stabilization of trunk and hip during standing by transferring the forces from the stance leg to the pelvis with an improved the femoral pelvic stability. In normal, healthy individuals, an average of 5 °of hip adduction angle was reported from double leg stance to single leg stance [46] since the hip abductors are an important pelvic stabilizer by resisting the varus torque of femur and enhance the femoro-pelvic stability during SLS [17, 18].

The measurement of knee extension strength showed a significant difference for both the groups at 3 months and preserved until 1 year, and NPRS has shown significant changes at all periods in both the groups. The improvement in quadriceps strength and a reduction in pain intensity is imperative which could have possibly enhanced the better physical function following TKR.

Significant improvement in hip abductor strength at 3 months and 1 year in HAS group was seen when compared to KS group. The participants with strong hip abductor strength walked further in SMWT at 1 year and performed better on SLS test at 3 months and 1-year duration. Considering the improvement observed in the HAS group it is most likely that the hip abductor would play a major role on the physical function measures following TKR. We recommend that the future randomized controlled trials should address the effectiveness of hip abductor strengthening following TKR with larger sample size.

## CONCLUSION

Hip abductor strengthening showed superior improvements in single leg stance test at 3 months and one year duration. Participants with strong hip abductors walked further in six minute walk test at one year duration when compared to the knee strengthening alone. Thus hip abductor strengthening exercises could be the potential contributors for improving physical function following TKR.

## Figures and Tables

**Table 1:** Baseline characteristics of participants.

Variables	Hip abductor strengthening group Mean (SD) or N (%)	Control group Mean (SD) or N (%)	p value
Age in years	63.3 (5.4)	62.8 (5.9)	0.962
Sex M: F	6 (60%): 4 (40%)	4 (40%): 6(60%)	0.913
BMI	26.5 (3.2)	29.8 (3.2)	0.618
Knee strength in pounds	36.1 (8.4)	34.8 (4.9)	0.060
Hip Abductor strength in pounds	36.1 (6.0)	36 (7.0)	0.537
SMWT in meters	255.1 (79.2)	201.1(64.4)	0.597
TUG in meters	16.3 (4.2)	18(4.3)	0.538
SLS in sec	8.3 (4.5)	6.5 (2.5)	0.355
NPRS	7.1(.56)	6.5(1.5)	

**Table 2:** Within group analysis of outcome measures.

Outcome measures	Baseline	1 month	3 month		1 year	Baseline to 1 month Mean (95% CI)	p value	Baseline to 3 month Mean (95% CI)	p value		Baseline to 1 Year Mean (95% CI)	p value
**Hip abductor strengthening group**												
Knee strength	36.1(8.4)	36.4(8.8)		40.9(10.1)	41.7(7.5)	.3(-.71-1.3)	<0.520	4.8(2.6-6.9)	<0.001	5.6(2.7-8.4)		<0.002
Hip abductor strength	36.1(6.0)	40.3(7.2)		42.6(6.9)	45.7(6.7)	4.2(2.4-5.9)	<0.001	6.5(4.1-8.8)	<0.001	9.6(7.8-11.3)		<0.001
SMWT	255.1(79.2)	304.6(108.6)		387.6(119.5)	474.1(160.5)	49.5(-8.1-107.1)	<0.084	132.5(62.6-202.3)	<0.002	219(104.1-333.8)		<0.002
SLS	8.3(4.5)	6.6(3.4)		13.7(5.4)	15.2(5.9)	-1.7 (-4.1-.75)	<0.271	5.4(2.5 -8.2)	< 0.001	6.9 (4.6-9.1)		< 0.001
TUG	16.3(4.2)	17.5(3.9)		13.3(4.0)	11.3(2.2)	-1.2(-3.9-1.5)	<0.347	3(.78-5.2)	<0.013	5(2.1-7.8)		<0.003
NPRS	7.1(.56)	4.2(1.1)		1.7(.6)	1.4(.9)	2.9(1.9-3.8)	<0.001	5.4(4.7-6)	<0.001	5.7(4.9-6.4)		<0.001
**Control group**												
Knee strength	34.8(4.9)	37.5(4.4)	42.3(4.3)		42.8(3.1)	2.6(-.53-5.7)	<0.070	7.5(6.3-8.6)	<0.001		8(5.6-10.3)	<0.001
Hip abductor strength	36(7)	37.12(5.8)	37.3(6.4)		39.8(6.2)	1.1(-.89-3.1)	<0.229	1.3(.38-2.3)	<0.014		3.8(2.8-4.9)	<0.001
SMWT	201.1(64.4)	216.8(90.5)	319.3(134.8)		380.6(124.7)	15.7(-30.3-61.8)	<0.445	118.2(35.3-201.1)	<0.012		179.5(90.5-268.4)	<0.002
SLS	6.5(2.5)	5.3(1.1)	7.62(2.7)		9.5(2.3)	-1.1(-4.8-2.6)	<0.100	1.1(-2.3-4.5)	<0.100		3(.3-5.6)	<0.027
TUG	18(4.3)	18.7(5.3)	15.2(4)		13.8(2.6)	-.75(-6.1-4.6)	<0.754	2.7(-.58-6.0)	<0.092		4.1(1.5-6.7)	<0.007
NPRS	6.5(1.5)	3.6(1.5)	2.1(.8)		1.1(.8)	2.8(1.4-4.3)	<0.002	4.3(2.8-5.9)	<0.001		5.3(3.9-6.7)	<0.001

SMWT- Six minute walk test, measured in meters; TUG- Timed up and Go test , measured in seconds; SLS- Single leg stance test, measured in seconds; NPRS- Numeric pain rating scale ; Knee strength ( quadriceps strength) in pounds; Hip abductor strength in pounds.

**Table 3:** Between group analysis of outcome measures.

Outcome measures	Baseline to 1 month Mean (95% CI)	p value	Baseline to 3 month Mean (95% CI)	p value	Baseline to 1 Year Mean (95% CI)	p value
**Between group analysis**								
Knee strength	-2.3(.5-.4)	<0.040	-2.9(-5.3 - -.45)	<0.023	-3.2(-6.5-.18)	<0.062
Hip abductor strength	3.0(.6-5.5)	<0.018	4.0(1.2-6.7)	<0.005	5.4(3.3-7.5)	<0.001
SMWT	19(-29.4-68.3)	<0.410	53.6(-10.4-117.7)	<0.095	88.3(36.5-140.2)	<0.002
SLS	-.82(-3.4-1.7)	<0.511	4.2(1.5-6.9)	<0.004	3.9(1.8-5.9)	<0.001
TUG	-.45(-5.6-4.7)	<0.857	.25(-3.2-3.7)	<0.883	.77(-2.9-4.4)	<0.661
NPRS	0.025(-1.4-1.5)	<0..972	1.02(-.4-2.4)	<0.148	-.72(-2.4-.86)	<0.330

SMWT- Six minute walk test, measured in meters; TUG- Timed up and Go test , measured in seconds; SLS- Single leg stance test, measured in seconds; NPRS- Numeric pain rating scale ; Knee strength ( quadriceps strength) in pounds; Hip abductor strength in pounds.

## APPENDIX-1

### Rehabilitation Following Total Knee Replacement (Knee Strengthening -Control Group)

#### Postoperative Day 1

Bedside exercises: ankle pumps, quadriceps sets, gluteal sets, hip abduction (supine), short-arc quads, straight-leg raise (if able with knee brace on).

Bed mobility and transfer training (bed to/from chair) Gait training with assistive device on level surfaces and functional transfer training (eg, sit to/from stand, bed mobility).

#### Postoperative Day 2

Exercises for active ROM, active assisted ROM, Knee ROM heel slides. Strengthening exercises (eg, ankle pumps, quadriceps sets, gluteal sets, heel slides, straight-leg raises) 1-3 sets of 10 repetitions for all strengthening exercises, twice daily.

#### Postoperative Day 3- 7(or on Discharge to Rehabilitation Unit)

Progression of ROM with active assisted exercises as necessary. Progression of strengthening exercises to the patient’s tolerance, 1-3 sets of 10 repetitions for all strengthening exercises twice/daily. Progression of ambulation distance. Progression of activities-of-daily-living training for discharge to home

#### Modalities

Ice 2-3 times per day, with lower extremity elevated for 20-30 min.

### Day 8 – Week 4

#### Range of Motion

Active assisted ROM for knee flexion, sitting or supine, using other lower extremity to assist Knee extension stretch with manual pressure (in clinic) or weights (at home)

#### Strength

Quad sets, straight leg raises (without knee extension lag), hamstring curls (standing), sitting knee extension, terminal knee extensions from 45° to 0°, step-ups (5- to 15-cm block), wall slides to 45° knee flexion, 1–3 sets of 10 repetitions for all strengthening exercises.

Criteria for progression: exercises are to be progressed (eg, weights, step height, etc.) only when the patient can complete the exercise and maintain control through 3 sets of 10 repetitions.

#### Pain and Swelling

Ice and compression as needed.

#### Functional Activities

Ambulation training with assistive device, as appropriate, with emphasis on heel strike, push-off at toe-off, and normal knee joint excursions.

Emphasis on heel strike, push-off at toe-off, and normal knee joint excursions when able to walk without assistive device.

Stair ascending and descending step over step when patient has sufficient concentric/eccentric strength.

### Outpatient Physical Therapy Advanced Strengthening Program Semi-Independent Phase (week 5 - week 12).

#### Supervised Physical Therapy Sessions of 2 Days a Week with Treatment Duration of 45-60 Minutes Warm-up (10-15 min)

Seated or supine knee AROM (flexion and extension). Alternated ankle dorsiflexion and plantar flexion. Passive knee extension stretch. Patellar mobilizations

#### Specific Strengthening (10-15 min), 1-3 sets of 10 Repetitions

Add 1 to 2-kg weights to the exercises for appropriate. Quadriceps sets. Straight-leg raises (goal to perform without a knee extension lag). Standing leg curls. Seated knee extension. Terminal knee extension from 45° to 0°

The specific strengthening exercises, performed in a supine or seated position, consisted of maximal isometric pain-free contractions (knee extensors and flexors), at different angles of knee flexion, and dynamic (concentric-eccentric) contractions against gravity. The isometric exercises were performed at multiple angles at 0, 45 and 90 degrees.

#### Functional Exercises (10-15 min)

Step-ups (forward and lateral), 5-15 cm, or climbing a flight of stairs with support. Sit-to-stands. Ambulation training with or without assistive device, as appropriate, with emphasis on heel strike, push-off at toe-off, and normal knee joint excursions. Stair ascending and descending step over step when patient has sufficient concentric/eccentric strength

#### Endurance Exercises (5-10 min)

Walking.

#### Cool-Down (5 min)

Ice and compression as needed, gentle stretching and ROM.

#### Incision Mobility

Soft tissue mobilization until incision moves freely over subcutaneous tissue.

#### Criteria for Progression

Voluntary quadriceps muscle control or 0° knee extension lag. AROM 0° to greater than 105° of knee flexion. Minimal to no pain and swelling. Exercises are to be progressed once the patient can complete 3 sets of 10 reps of the exercise correctly and feels maximally fatigued.

## APPENDIX-2

### Rehabilitation following Total Knee Replacement (Hip Abductor strengthening group)

#### Postoperative day 1

Bedside exercises: ankle pumps, quadriceps sets, gluteal sets, hip abduction (supine), short-arc quads, straight-leg raise (if able with knee brace on).

Bed mobility and transfer training (bed to/from chair). Gait training with assistive device on level surfaces and functional transfer training (eg, sit to/from stand, bed mobility).

#### Postoperative day 2

Exercises for active ROM, active assisted ROM, Knee ROM heel slides.

Strengthening exercises (e.g., ankle pumps, quadriceps sets, gluteal sets, heel slides, straight-leg raises, supine hip abduction) 1-3 sets of 10 repetitions for all strengthening exercises, twice daily.

#### Postoperative day 3- 7(or on discharge to rehabilitation unit)

Progression of Hip abduction exercises in supine and standing. Progression of ROM with active assisted exercises as necessary. Progression of strengthening exercises to the patient’s tolerance, 1-3 sets of 10 repetitions for all strengthening exercises twice/daily. Progression of ambulation distance. Progression of activities-of-daily-living training for discharge to home.

#### Modalities

Ice 2-3 times per day, with lower extremity elevated for 20-30 min.

### Outpatient Physical Therapy Specific Strengthening Programme (Day 8- week 4)

#### Goals

Increase range of motion (ROM). Decrease edema and pain. Gait training. Independence with activities of daily living (ADLs).

#### Exercises

Seated or supine knee active range of motion (AROM). Alternated ankle dorsiflexion and plantar flexion. Quadriceps sets, Straight-leg raise, Hamstring sets, seated knee extension. Supported single standing for balance. Repeated sit-to-stand transfer training. Ambulating with appropriate assistive device (walker).

#### Hip Abduction Exercises

Abduction in side lying, unilateral hip abduction performed in standing, isometric hip abductor strengthening exercise, Calm exercises for hip abductors.

#### Modalities

Ice 2-3 times per day, with lower extremity elevated for 20-30 min.

### Ę Criteria for Progression to Phase III

AROM approaching 100-120° of knee flexion, minimal pain/swelling.

Independence in mobility in and out of the home.

### Supervised Physical Therapy Sessions of 2 Days a Week with Treatment Duration of 45-60 Minutes Warm-up (10-15 min)

Seated or supine knee AROM (flexion and extension). Alternated ankle dorsiflexion and plantar flexion. Passive knee extension stretch. Patellar mobilizations.

### Specific Strengthening (10-15 min), 1-3 Sets of 10 Repetitions

Add 1 to 2-kg weights to the exercises for appropriate. Hip abductors with weights in supine and standing. Quadriceps sets. Straight-leg raises (goal to perform without a knee extension lag). Standing leg curls. Seated knee extension. Standing terminal knee extension from 45° to 0°.

The specific strengthening exercises, performed in a supine or seated position, consisted of maximal isometric pain-free contractions (knee extensors and flexors), at different angles of knee flexion, and dynamic (concentric-eccentric) contractions against gravity (hip abductors). The isometric exercises were performed at multiple angles, because strength gains are known to be specific to the trained positions.

### Hip Abduction Exercises

Abduction in side lying, abduction in standing, side walking, isometric contraction of hip abductors. Unilateral hip abduction performed in side lying with the use of ankle cuff weights (3 sets of 10 at a 10 RM resistance). Unilateral hip abduction performed in standing with the use of ankle weights (3 sets of 10 at a 10 RM resistance). Standing wall isometric hip abduction. Calm exercises with resistance on theraband. Performed in unipedal stance with the opposite limb in 90 degrees of hip and knee flexion (3 sets of 10 with 5 second holds).

### Functional Exercises (10-15 min)

Step-ups (forward and lateral), 5-15 cm, or climbing a flight of stairs with support. Sit-to-stands. Ambulation training with or without assistive device, as appropriate, with emphasis on heel strike, push-off at toe-off, and normal knee joint excursions. Emphasis on heel strike, push-off at toe-off, and normal knee joint excursions when able to walk without assistive device. Stair ascending and descending step over step when patient has sufficient concentric/eccentric strength.

### Endurance Exercises (5-10 min)

Walking.

### Cool-down (5 min)

Ice and compression as needed, Gentle stretching and ROM.

### Incision Mobility

Soft tissue mobilization until incision moves freely over subcutaneous tissue.

### Criteria for Progression

Voluntary quadriceps muscle control or 0° knee extension lag. AROM 0° to greater than 105° of knee flexion. Minimal to no pain and swelling. Exercises are to be progressed once the patient can complete 3 sets of 10 reps of the exercise correctly and feels maximally fatigued. Participants will perform exercises weekly once or twice (continue all exercises, 1-3 sets at 10-20 repetition) after 12 weeks.They were asked to continue their home based exercise and the exercise adherence was ensured through logbook and periodic telephone calls.
